# Pictorial depiction on controlling crowd in smart conurbations using Internet of Things with switching algorithms

**DOI:** 10.1038/s41598-024-61993-5

**Published:** 2024-06-02

**Authors:** Hariprasath Manoharan, Osamah Ibrahim Khalaf, Sameer Algburi, Habib Hamam

**Affiliations:** 1 Department of Electronics and Communication Engineering, Panimalar Engineering College, Poonamallee, Chennai, 600 123 Tamil Nadu India; 2 Department of Solar, Al-Nahrain Research Center for Renewable Energy, Al-Nahrain University, Jadriya, Baghdad, Iraq; 3 Al-Kitab University, Kirkuk, 36015 Iraq; 4 Université de Moncton, Moncton, NB E1A3E9 Canada; 5 School of Electrical Engineering, University of Johannesburg, Johannesburg, 2006 South Africa; 6 Faculty of Engineering, Université de Moncton, Moncton, NB E1A3E9 Canada; 7 Hodmas University College, Taleh Area, Mogadishu, Somalia; 8 Bridges for Academic Excellence, Tunis, Tunisia

**Keywords:** Crowd management, Internet of Things (IoT), Image processing, Switching network, Engineering, Energy infrastructure

## Abstract

The proliferation of smart conurbations entails an efficient system design for managing all the crowds in public places. Multitude controlling procedures are carried out for controlling compact areas where more number of peoples is present at several groups. Therefore for controlling purpose the proposed method aims to design a pictorial representation using Internet of Things (IoT). The process is carried out by taking images and then organizing it using switching techniques in the presence of square boxes where entire populace is identified on real time experimentations. For processing and controlling the occurrence a separate architecture is designed with analytical equivalences where all data set is stored in cloud platform. Further the incorporation of system model is carried out using Switching Based Algorithm (SBA) which adds more number of columns even for high population cases. In order to verify the effectiveness of proposed model five scenarios are considered with performance evaluation metrics for SBA and all the test results provides best optimal results. Moreover the projected model is improved with an average percentage of 83 as compared to existing models.

## Introduction

The problem of multitude management technique which is considered as a usual process by several forces needs some of the advanced technologies for controlling purpose. Most of the systems that are designed for monitoring multitude can able to detect few people in the public places. Even though many systems are designed with powerful camera structures the procedure of classification and pre-processing remains un-established. However during such investigations it is found that Internet of Things (IoT) with image processing technique proves to be better method for monitoring more number of crowd in any public places or buildings. In addition many government and non-government agencies prefers establishment of IoT in their work stations for managing and accumulating multitudes in some places. The contextual information that is shared in IoT procedure is that access from different locations can be enables within short period of time. Additionally it is possible to connect *n* number of devices with additional security features in many relevant smart accessing expedients. Moreover in IoT procedure the problem of congestion can be avoided completely using loop framework model that observers different situations using removal of unwanted components in the system this transmission devices can be reduced in the integration process.

**Figure 1:**
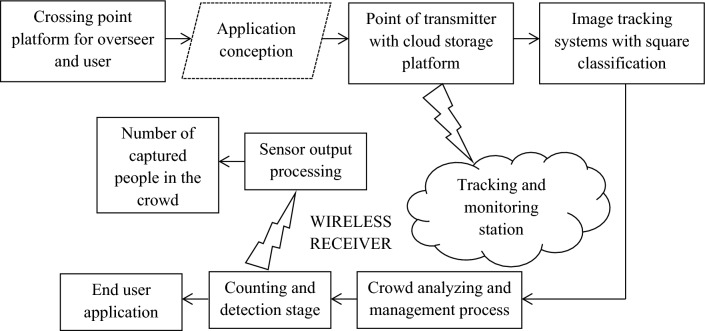
Block diagram of crowd management system.

In the proposed method it can be observed that various types of intelligence such as human, artificial and collective procedures are combined to collect information in a useful way. Due to presence of more number of individuals it is necessary for an identification method that combines the above mentioned intelligent techniques even in presence of different switching characteristics. Further to maximize operational efficiency in smart cities under various sequence as no order of arrangements are made and the process is considered with distribution techniques it is much difficult to identify whole set of individuals if more crowd is present without any limitations. Therefore the collective strategies with distinct intelligence with agglomerations is combined with IoT where by identifying core infrastructure it is possible to manage the presence of crowd in an effective way. Further the IoT process contains communication modules that provides fast access link for providing communication between one machine to another machine that comprises of futuristic standards. Figure 1 portrays the block diagram of crowd management system using IoT process that starts with crossing point identification of both administrator and corresponding user. This procedure of integration is termed as application conception that provides quick point of transmission using secured cloud storage platform. In the storage device many images are stored and it is used as reference for identifying number of identical people crossing the same area. The above mentioned storage platform is followed by tracking systems that manages entire crowd by informing the people crossing *n* number of times. In addition a separate wireless monitoring station is used for processing the sensor layer output to all end users. The information is passed using a wireless receiver using counting and detection stages and finally it will be displayed in the designed applications. Moreover the above mentioned block diagram that is implemented for design purpose reduces higher complexities in all surrounding areas which in turn minimizes the group formation thus enabling better healthy environmental conditions.

### Literature survey

The process of exploration using IoT twitches by observing all the existing models as the same process needs to be elevated with new and novel design paradigms. Therefore it is essential to have complete knowledge on existing methods regarding crowd management process. Usually the crowd management is carried out by directly implementing the design without any representations that are relevant to mathematical models. But some of the researchers have formulated the basic representations that support to build the entire process with step-by-step procedures. In^1^ a dynamic mode of operation is carried out to control the crowd where a feedback technique is enabled in all smart cities and even at rural areas. The dynamic operation is examined under various scenarios that provide support to three different applications such as transportation, shopping and disaster management. All the above mentioned applications can be controlled by automated mode of operation thus the accomplishment of feedback procedures is observed as undesirable component in the entire system. For carrying out automatic operations a Lagrangian model is proposed^2^ that switch all the random process in the crowd from large sector areas. The Lagrangian mathematical representations are specially designed for perambulators using two dimensional representation where acceleration segments are completely avoided. But the major drawback in the system design is that when entire crowd is monitored both position and acceleration parameters are much important as they are considered as external force measurements.

Apart from smart cities there are some multitudes that exist in buildings^3^ and a wireless module is designed in such cases for monitoring it for long range distance. Even though the wireless module ranges high it is not essential to implement it in smart building as short range modules with much lower cost exists in the market. Though the implemented long range module is used in smart buildings it augments artificial intelligence as an advanced technique thus making all the nearby places to be examined under same device without any variations. In addition all the physical movements are monitored using a partial derivative equation that uses a separate catastrophe theorem for predicting the necessary solutions under emergency conditions^4^. The benefit of derivative model is that it is used for monitoring residents in highly density areas where all critical points are focused using square shaped structures. Yet the density model is designed using one dimensional incident that fails to observe all the surrounding users in entire crowd. The above mentioned one dimensional model is extended in a moving platform in order to monitor all lively nature of foot bridges where most of the people usually gathers^5^. The technique of sentient measurements are carried out under a special condition which is termed as motionless interaction areas. Even though many areas are monitored with zero motion conditions there are some special cases that use adjacent trembling process with human induced vibrations. Instead of using peripheral vibrations the controlling procedures can also be implemented using IoT platform^6^ where an intelligent detection model is created for making all the members to function in an effective way. Additionally simulation results are carried out using a separate communication unit that enables the users to manage all types of difficulties in expanded network areas. Conversely if the network is expanded to sixth generation the bandwidth and other specifications needs to be modified thus making entire system to be re-designed in such conditions.

The traces of crowd in public management system is monitored using flow rate of wireless modules^7^ where all the system data is counted. In this counting stage it is not possible to predict the number of human interference as no explanation on such procedures is provided. On the other hand the process is exploited using human interface technique that supports the regressive models based on automatic management procedures. Furthermore with regression model neural networks are established that predicts the entire flow rate in the wireless modules. Some of the researchers in Arabic countries^8^ created a unique model for crowd detection and management procedures using the hidden unit in the form of images with IoT. A new architecture is framed using existing data set and it is compared with current values in the entire network where security problems can be easily deciphered. Though security constraints are enhanced a surveillance camera must be connected for detecting all live pictures in the public and in this case it is converted to open problematic case. With additional bounded domains a theoretical framework is processed using kinematic representations that checks the geometrical domain if IoT procedures are implemented^9^. The aforementioned geometrical representations are made by distributing the weights and parameters to the crowd only in exist zone regions. Due to such distributions only certain zones are monitored whereas other conditional zones are left unloaded. Consequently to monitor all zone regions edge computing procedures are determined with scheduling measures^10^ using low powered devices where low resources are allocated in the system. Since only few resources are allocated the efficiency of the device cannot be improved if more crowd is present in some places. A potential method on sensing crowd with potential limitations is established^11^ using data analytics technique where it is possible to forecast all the complex urban environments without any delay. Such advantages in the system provide high safety thus making the data to be integrated in near future. Some of the future advances for developing the model are discussed using virtual reality procedures^12–15^. Thus by considering all the above mentioned developments efforts are made in constructing a new system model with better optimization techniques that are discussed in subsequent sections and the comparison with existing works are provided in Table 1.

**Table 1:** Existing vs. proposed.

References	Methods/algorithms	Objectives			
		A	B	C	D
^16^	Collaborative edge computing for crowd management	✓		✓	
^17^	Maximum polling for crowd localization with supervised algorithm		✓		✓
^18^	Geographic characteristic analysis for crowd identification in urban areas			✓	✓
^19^	Risk identification for physical contacts using dynamic analysis	✓			✓
^15^	Mass crowd management with radio frequency identifications		✓	✓	
Proposed	Switching based algorithms with Internet of Things for crowd identification and control	✓	✓	✓	✓

A: IoT transmissions and dynamic features; B: identification of flow rates; C: data lag periods; D: data loss.

## System model for crowd management

Since a fixed infrastructure is present in all public places a large amount of crowd is much difficult to handle thus creating complex environments where more individuals suffers with exhaustion problems. In addition even if adequate planning procedures are made there is more change of disaster of overcrowding occurs is fixed cases. It is also observed that in fixed environments if more number of individual gathers then the amount of risk will be much higher where both prevention and management cannot be processed without introducing advanced techniques and intelligent screening process. Moreover different methods are even followed to reduce the crowd with appropriate planning procedures where every individuals moves from side-to-side but at the same time if density increases then a challenging condition exists. Therefore for prevention cases IoT procedures can be followed for better identification in presence of confined spaces. The problem formulation using IoT determines the type of design to be installed for managing the entire multitude in either small or big area platforms. Usually an analytical model is designed for integrating the hardware and loop formation with coding integrations. In the proposed method, the formulations are designed using image processing (involves both pre and post processing steps) where entire image is captured using highly classified cameras. Additionally the projected model monitors large number of crowds in public gatherings thus dynamic variations will be noted for each variations with respect to time periods. Thus the dynamic variations is formulated using Eq. (1) as follows,

1 $${dv}_{i}=\sum_{i=1}^{n}{m}_{i}*{o}_{i}$$

where,

${m}_{i}$ and ${o}_{i}$ denotes poignant and overall number of individuals in the entire crowd.

Equation (1) denotes the increase in dynamic variations which is represented using composite matrix. But the overall multitude in the entire area is represented in the form of transmission function which is used for converting composite matrix to representations in time domains. During this conversion process an alarm will be indicated to make the individuals shift from more crowded to uncrowned areas which are indicated using device formulations as represented in Eq. (2).

2 $${c}_{i}=max\sum_{i=1}^{n}\frac{{\tau }_{i}*{d}_{in}}{{TM}_{i}}$$

where,

${\tau }_{i}$ indicates the association between different types of devices, ${d}_{in}$ represents variations in type of different devices, ${TM}_{i}$ describes total number of members in the crowd that is recorded in the communicating devices.

Equation (2) is established as first objective function that is used for maximizing the association matrix. The association matrix provides a set of relationship where only captured people are represented with transformation values. However there are few number of peoples where the camera fails to capture the images and a separate active and downcast time period intervals are represented for providing differentiation between the two cases as follows,

3 $${f}_{a-d}\left(i\right)=min\sum_{i=1}^{n}\frac{{t}_{a}+{t}_{d}}{{t}_{in}}$$

where,

${t}_{a}$, ${t}_{d}$ and ${t}_{in}$ denotes active, downcast and total time period representations.

Equation (3) denotes second objective function which is used for minimizing flow rate of representations in two different phases. Since many users are represented in the open raised area there will be some time period of delay to shift from one place to another. This delay is denoted as lag time and is represented using Eq. (4) as follows,

4 $${lag}_{i}=min\sum_{i=1}^{n}\frac{{m}_{i}(t)}{{\sigma }_{t}(i)}$$

where,

${\sigma }_{t}$ represents the time period of shift.

The time period of shift must be minimized in order to control the dead rate of components. Thus to prevent this loss function is defined using Eq. (5) as follows,

5 $${loss}_{i}=min\sum_{i=1}^{n}\frac{{out}_{i}\left(t\right)*{G}_{i}}{N}$$

where,

${out}_{i}$ represents the output of shifting periods, ${G}_{i}$ indicates the number of pulverized veracity segments, $N$ describes total number of iterations.

The pulverized veracity segments are measured for each object with respect to location of pixel values which is formulated using Eq. (6) as follows,

6 $${gt}_{i}=\sum_{i=1}^{n}\left({pr}_{i}-{po}_{i}\right)*{\vartheta }_{i}$$

where,

${pr}_{i}$ and ${po}_{i}$ denotes reference and original pixel values respectively, ${\vartheta }_{i}$ indicates total number of nearest neighbors.

Thus the objective function is represented by combing the minimization and maximization functions from Eqs. (1)–(6) as follows,

7 $${obj}_{i}=min\sum_{i=1}^{n}{f}_{a-d}\left(i\right),{loss}_{i},{lag}_{i}, max\sum_{i=1}^{n}{c}_{i}$$

Equation (7) indicates a two objective case where minimization problems are subject to flow rate, loss and lag functions whereas maximization problems are subject to maximization of communicating devices. The abovementioned objective function is formed in a loop matrix that is integrated with optimization algorithm which is described in subsequent section.

## Optimization algorithm

**Figure 2:**
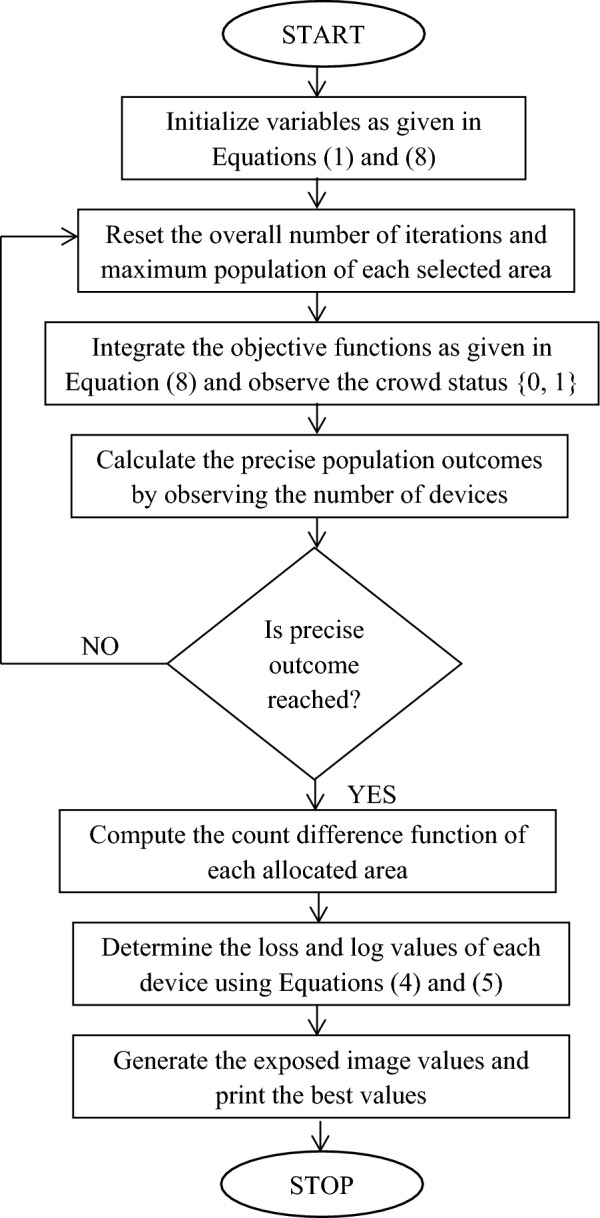
Flow chart of SBA for crowd management.

Since a computer based vision matrices with image set classifications are used for identifying the entire crowd in a large sectional area it is essential to improve the accuracy of detection using unit classification optimization. Therefore in the proposed technique a Switch Based Algorithm (SBA) is used in detection process using three distinct unit types. Even in the presence of different data transmission techniques SBA provides greater flexibility and adaptability over changing environments as large amount of crowd is sorted with proper identification with square boxes. An IoT monitoring device detects various individuals but at the same time more frame collisions can be observed within short period of time which makes identification process to remain at half complex modes. But with SBA and IoT processing units a straightforward identification mechanism can be followed thus frame collisions are avoided completely. The primary benefit of SBA is its ability to accommodate a greater number of columns for identification instances, ensuring accurate identification of the entire crowd in both the inward and outward directions. In addition, unlike previous techniques, this algorithm incorporates a density support matrix into the column vector, which accurately represents the local changes throughout the entire system^20–22^. In SBA, the input is pre-processed using an image set, while the output is not represented using a classifier set. This distinguishes SBA from other networking algorithms that monitor crowd size. Moreover three different layers are chosen and for all layers a special filter is used in order to capture all low scale segments. The integration of proposed formulation with optimization algorithm as shown in Fig. 2 is carried out using mathematical representations as follows,

8 $${P}_{i}=max\sum_{i=1}^{n}\frac{{\alpha }_{i}(t)}{{I}_{q}}$$

where,

${\alpha }_{i}$ indicates accuracy of all changing categories with time period, ${I}_{q}$ denotes the quality of image set.

Equation (8) represents the performance evaluation of SBA with respect to image quality factor. This the variation in count values is represented using difference Equation as follows,

9 $${count}_{i}=min\sum_{i=1}^{n}{count}_{p}\left(i\right)-{count}_{o}\left(i\right)$$

where,

${count}_{p}$ and ${count}_{o}$ indicates the expected and original count respectively.

From Eq. (9) is it indispensable that more variations in the count with respect to changing pixel values must be minimized. This minimization factor is subject to image axis determination case which is represented using Eq. (10) as follows,

10 $${F}_{i}=\sum_{i=1}^{n}I\left(x,y,z\right)+{\gamma }_{i}$$

where,

$I\left(x,y,z\right)$ denotes image representation matrix is three axis form, ${\gamma }_{i}$ represents additional moment frame in three axis.

The probability values of exposure time in *x,y,z* axis is represented using threshold values as it is a vital factor that all images must be exposed in large sectional area. This exposure image in SBA is represented using Eq. (11) as follows,

11 $${E}_{i}=\sum_{i=1}^{n}{d}_{c}\left(i\right)*\Delta {t}_{i}$$

where,

${d}_{c}$ indicates critical distance value that cannot be captured.

**Figure:**
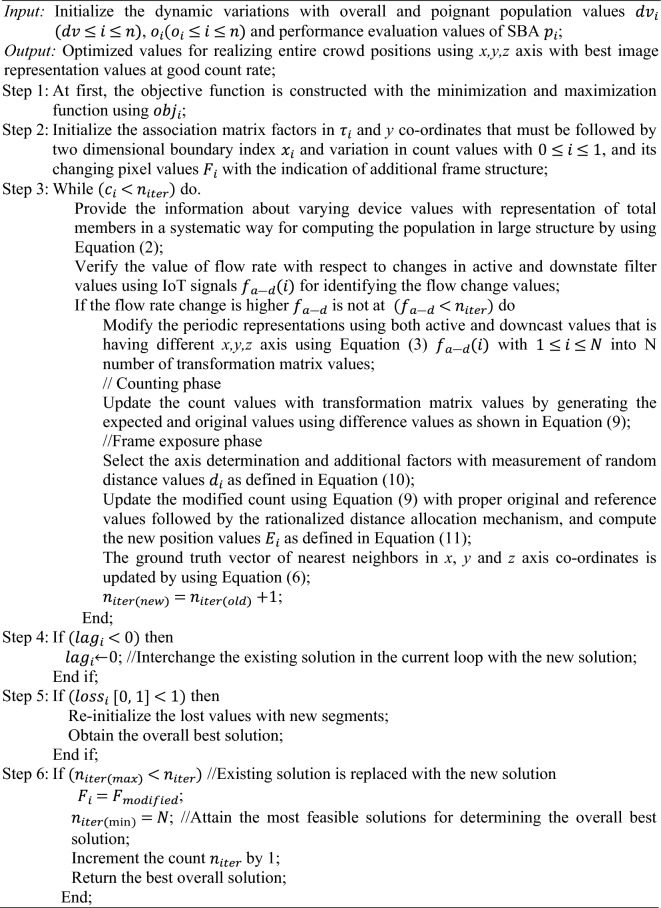
Algorithm—Switch Based Algorithm (SBA)

The aforementioned loop formation is implemented in software and if looping conditions are found to greater than expected number of iteration then loop process will continue until end state is reached. All the loop formation is simplified and it is mentioned using a separate flow chart that tracks the objective functions.

## Experimental results

This section examines the verification of IoT process for monitoring entire crowd in selected areas using image processing systems. Also the entire hardware setup is designed in such a way to test in a particular area whether the people in the public places can able to move to other locations in case of dynamic management systems. The designed formulations are applied in the hardware model where it is directly connected with simulation setup using MATLAB and the deviations are further noticed in accurate way. However in the construction design it is necessary to check the length of intervals as it varies to certain extent therefore a time domain analysis is created in the simulation setup. In case if there is large variation in parametric values then stability of the system is affected thus group of images is subject to disgust conditions. To avoid such conditions entire crowd is monitored at same time using square image boxes and if it crosses a certain limit then velocity of corresponding groups is decreased. For real time simulation results both the formulated system model and optimization algorithm is examined under the following scenarios.

Scenario 1: Monitoring dynamic variations.
Scenario 2: Maximization of transmission unit functions.
Scenario 3: Flow rate in the system.
Scenario 4: Minimization of lag functions.
Scenario 5: Performance evaluation of SBA.

In the proposed method the IoT setup with hardware components that are integrated with various modules such as processors (Raspberry pi), Portable cameras, Switch based cameras, wireless data transmission networks, internal and external power setup is converted to an equivalent representation where a data processing toolbox with IoT is represented using MATLAB. As indicated in Table 2 all data metrics is defined properly in MATLAB and a direct way of indication is made only for analyzing data outcomes thereafter indications are made with presence and absence of various population groups. For real time identification of growing population in limited boundary regions it is essential to implement complete smart assessments systems interconnected with edge computing models with centralized IoT infrastructure therefore in the proposed method Things speak application which indicates complete time loops are combined as direct representation procedure. Since Things speak is used for observing outcomes all visualizations in fixed infrastructure can be captured at instant time period whenever a new data is observed. The above mentioned direct representations can also be observed with individual web pages thus alerts are provided for overcrowding situations where necessary actions are generated without any delay. The data set for validation is observed with various groups that are clustered at different places where images are collected and processed from existing data set.

**Table 2:** Data metrics.

Parameters	Total number of initializations
Number of states	100
Number of measurements	17,800
Box particle filters	1470
Filter state vector	10
State extensions	245
Cluster radius	5.6 m
Number of individuals	1000
Switching time	15 s
Error deviations	0.1
Identification resolutions	640 × 480

**Figure 3:**
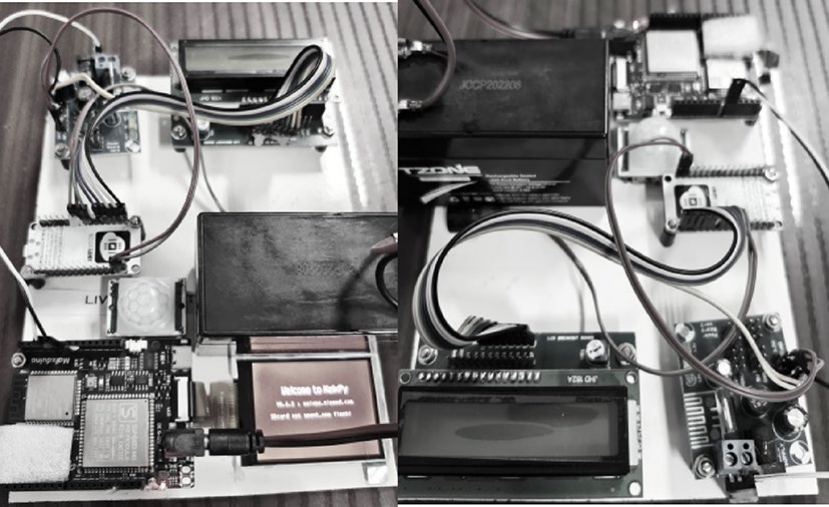
Implementation prototype for live crowd management systems.

However in the proposed method human participants are not involved for gathering data set whereas only the indications are made for 1000 people under 10 groups with the existence or nonexistence. Further no other information is collected even if existence state representations are made thus only square box indications will be provided in projected model. Table 2 indicates the data metrics for proposed method that is processed with simulation setup and the prototype for live crowd monitoring is illustrated in Fig. 3. All the five scenarios are carried out with different destination thus making entire velocity values of separate groups to be converged at similar positions. In case if the position varies then grouping factor will diverge without any fixed values. Therefore to avoid this situation velocity periods are noted thus providing best link and assessment to entire crowd sections with low network congestion problems. In the proposed method congestion rate is reduced for an amount of less than 42 percentage thus entire data set is added without any collision in the memory system. The detailed description and simulation outcomes are described for each scenarios as follows and the significance of scenarios are listed in Table 3.

**Table 3:** Significance of scenarios.

Scenarios	Importance
Monitoring dynamic variations	To indicate the changing states and orientations for overall population
Maximization of transmission unit functions	To observe better indications in transmission units in presence of large crowds
Flow rate in the system	To analyze immediate response on overcrowd situations in limited boundaries
Minimization of lag functions	To provide solutions for delayed responses and identification factors
Performance evaluation of SBA	To observe all changing category of images with switching algorithms

### Scenario 1

**Figure 4:**
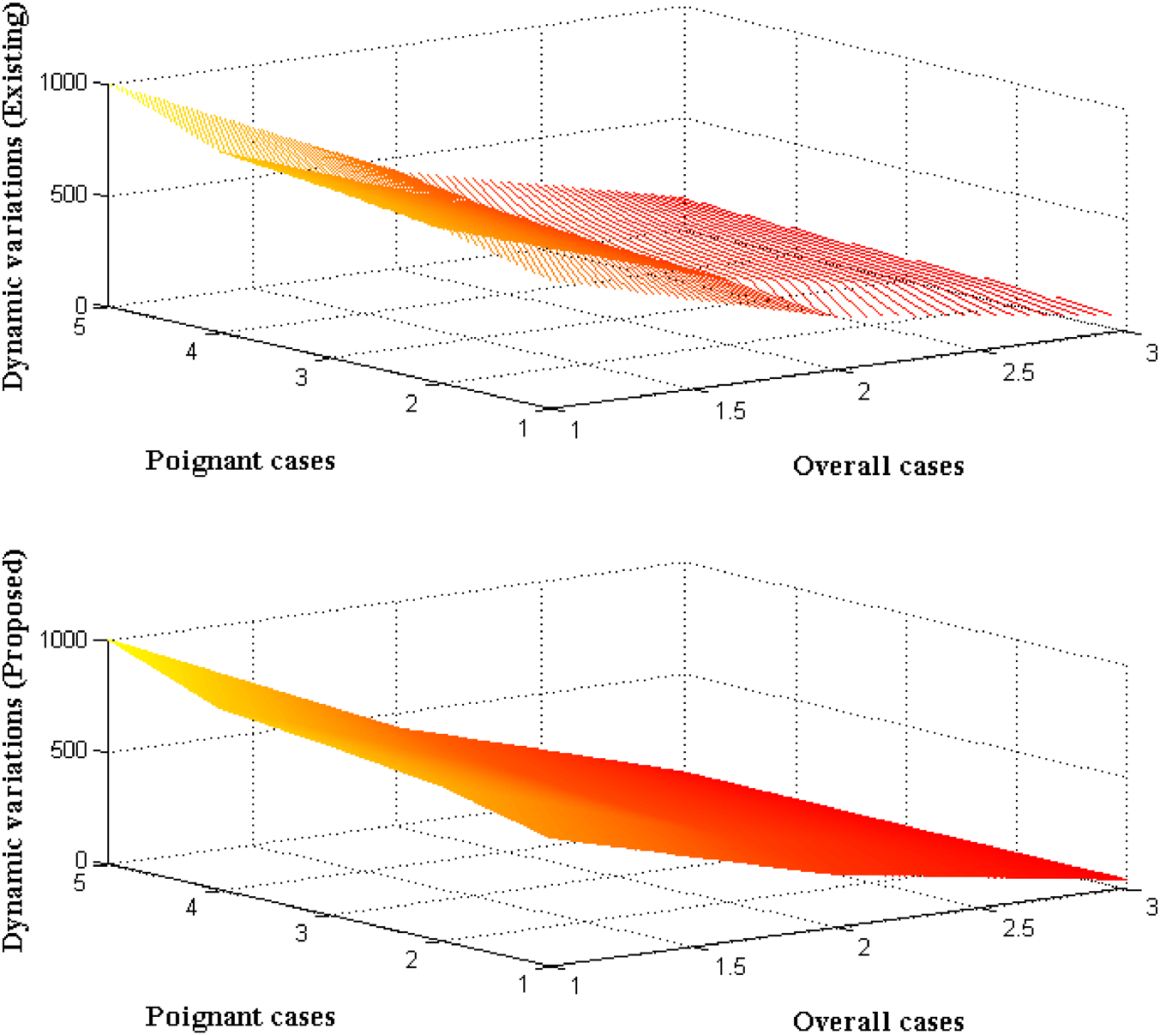
Dynamic variations in multitude.

The dynamic variations in the entire crowd are always a challenging phenomenon to be detected as variations usually occurs in a large area and it cannot be controlled. Therefore for monitoring purpose overall random population in a particular area is provided at earlier stage. This type of memory organization is usually carried out in crowd management process and it is termed as orientation procedure in IoT process. In this case images of every entities are taken and stored in the system in order to alert the time of presence in a particular locations. In this process a square box is used for indicating the time periods using green and red color marks where if a particular individual crosses the time limit then red mark is indicated. This restraint is provided to central monitoring station thus in turn alerting the individual to move from certain position. After observing the interval position changes in current locus is reproduced which provides destination values of dynamic movements in the crowd. The dynamic variations that are simulated with SBA are represented in Fig. 4.

From Fig. 4 it is pragmatic that five different overall populace is chosen from 500 to 1000 in a random variations. From the random variations only 35 to 40% of individuals are in diverse location whereas remaining individuals are in static positions. This static position makes the raise in alarming conditions which directly gives rise to dynamic variations in the system. These variations are compared with existing models^5^ with respect to time periods and it is detected that proposed method provides better dynamic behavior as compared to other methods. This can be proved for large population where 1000 number of individuals are present where for each variation the total corresponding period is equal to 56 s in case of projected method. With same population category existing method provides a variation of about 136 s which is much higher than expected values.

### Scenario 2

**Figure 5:**
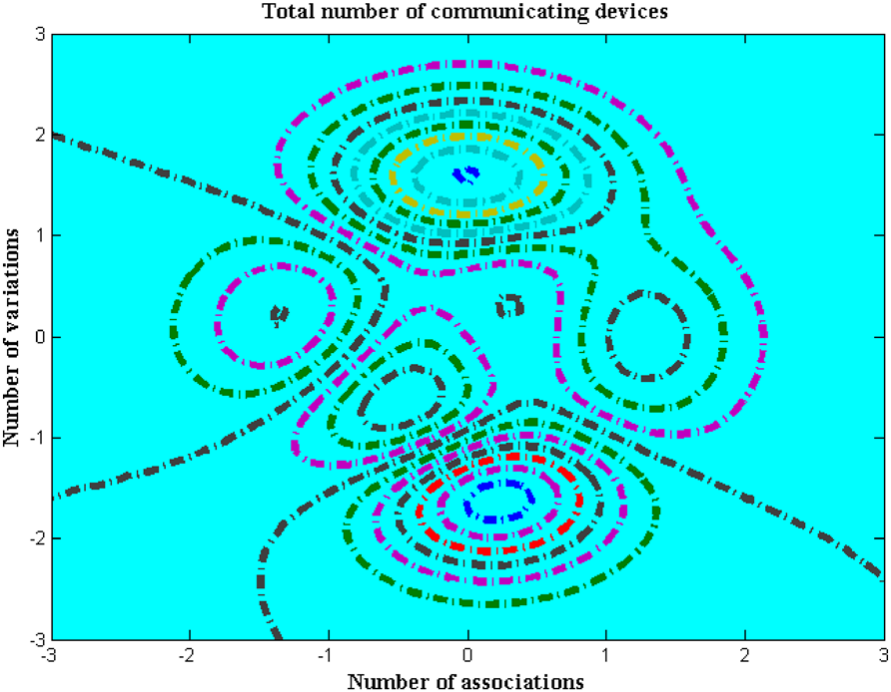
Communicating devices with variation factor.

The number of transmission units provides certain information for all the output cases that guarantees good association between the connected units. To establish a good communication unit it is essential to produce number of variations with respect to device connection. Thus in the proposed method for identifying crowds in the entire area some changes in the communication devices are made and established. Usually the above mentioned establishment is made by converting complex functions to time domain representations as for each time varying factor a set of values will be generated. Also different mode of devices will function effectively only when all individuals are captured separately therefore this scenario gives raise to two different association matrix which are allocated for captured and non-captured images. In the final transmission device representation it is required that maximization of transmission units must be processed only for captured images after separating it from total number of members in the entire multitude areas. Figure 5 indicates total number of communicating devices with variation factors.

From Fig. 5 it is realistic that proposed method captures all images in the crowd with changing communication and transmission unit. For experimental verification the number of good association after time domain conversion is considered as 12, 16, 24, 36 and 40 respectively. The five different association matrix are observed for variation factors of 2, 3, 5, 7 and 8 that constitute as a separate exemplification. During the above mentioned disparities number of communicating device for proposed method is lower as compared to existing method^5^. This can be demonstrated with big association matrix of 40 with 8 distinct discrepancies and during all these considered aspects the proposed method uses only 18 number of transmission unit from one end to other whereas existing method implements 46 different units. This in turn increases the complexity of existing method with more number of association matrix formations and hence it is avoided in proposed model.

### Scenario 3

**Figure 6:**
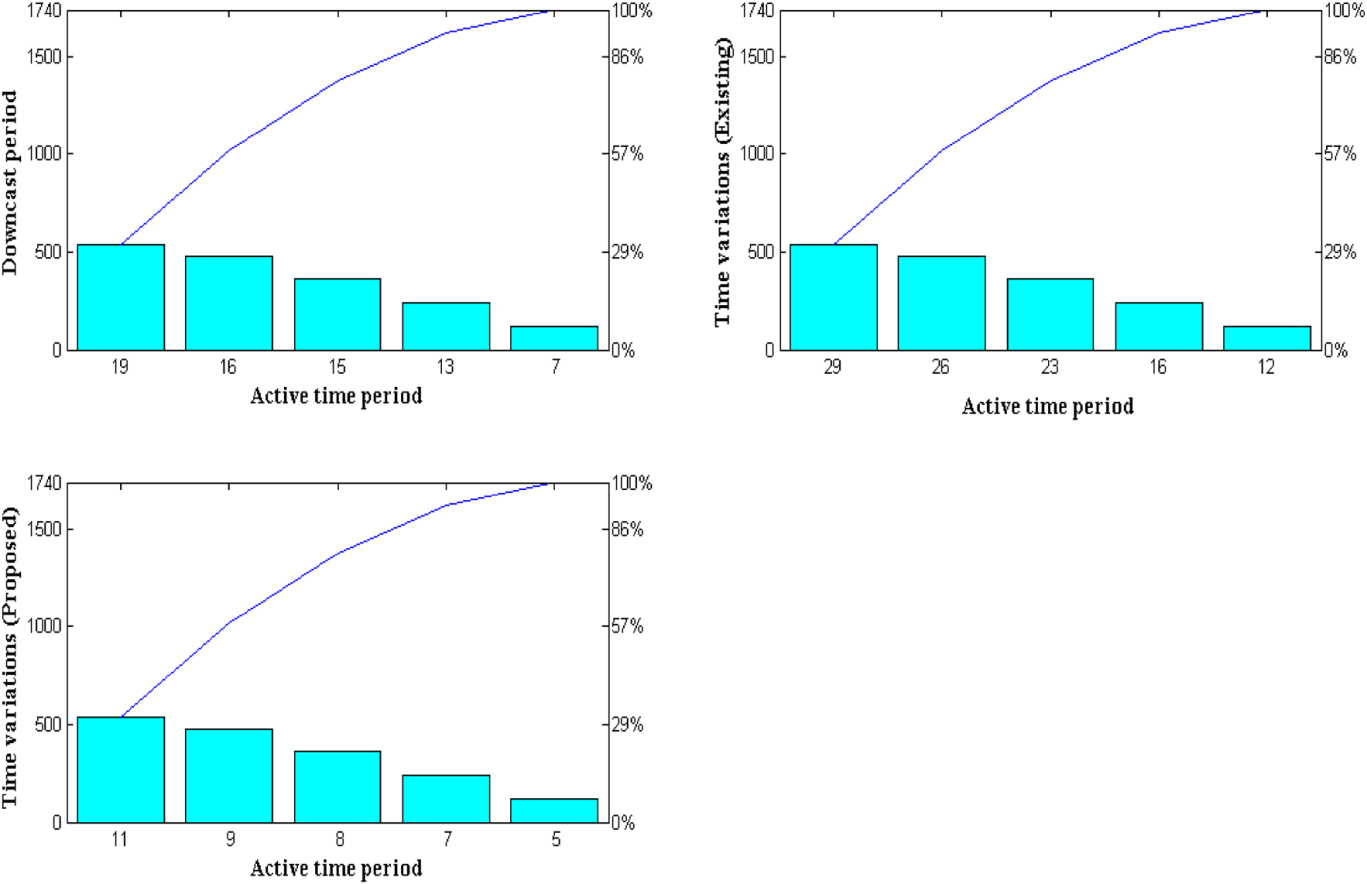
Variations in time periods.

After conversion of time domain representations three different time periods are chosen for determining the actual flow rate in the entire system. This type of scenario examination is carried out using active, downcast and total time period representations. The major reason for dividing such time period representation is that flow rate in the system must be minimized. In addition the flow rate is minimized for reducing the speeding up level of every individual in the crowd as their images are not captured in previous case. Due to inappropriate image capturing process two different time periods are added and it is separated from total time period as represented in Eq. (3). As a result of this separation flow rates are adjusted at proper proportion thus secondary conversions are made from worst association matrix to good time period representation values. The simulation results for determining the flow rate is explicated in Fig. 6 with optimized association matrix level.

From Fig. 6 it is detected that active time period representations are varied from 120 and later it is adjusted in distinct ranging types as 240, 360, 480 and 540 respectively. For each active time representation the downcast time in seconds is marked as 7, 13, 15, 16 and 19 which indicates that only small time periods are permitted for determining the amount of flow rates. During the above mentioned time period representation variations for both proposed, existing methods^5^ are simulated and compared. From the comparison outcome it is much clear that time variations are much lesser in proposed method as most of the individuals in the multitude are captured correctly. This can be verified with active and down cast time periods of 480 and 16 s where during these periods the flow rate is much lesser for about 9 s in case of proposed method. With same variations in time periods existing method provides a flow rate of about 26 s and it is much higher than expected.

### Scenario 4

**Figure 7:**
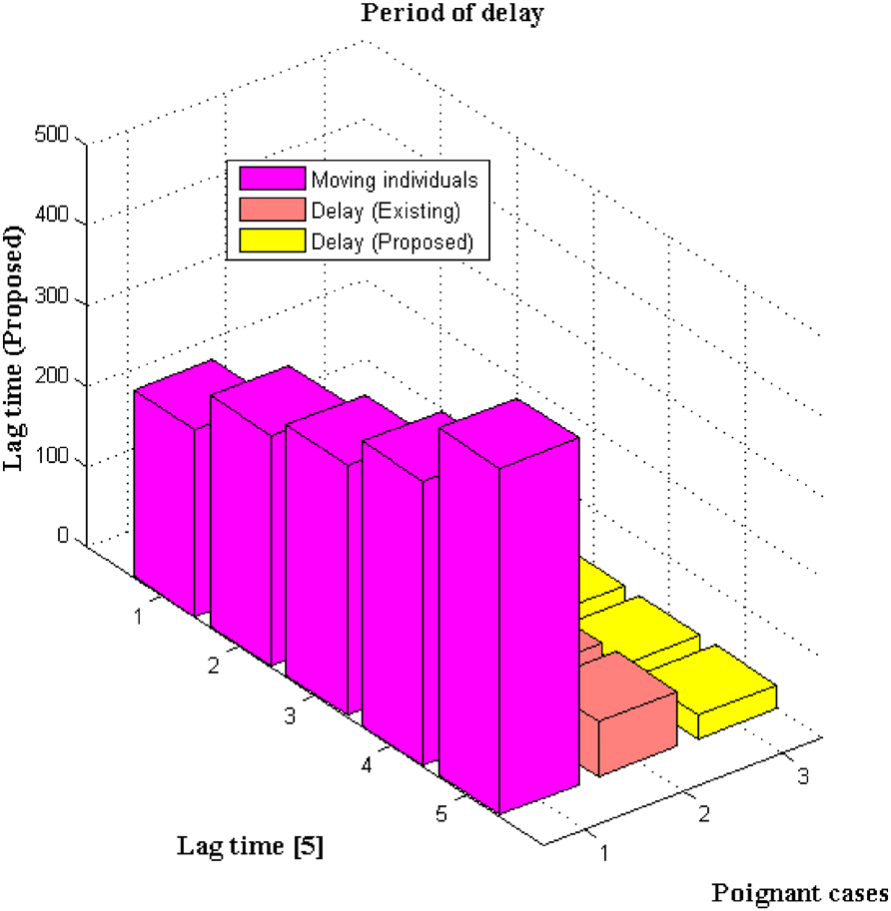
Comparison of lag time.

In the operative period of crowd monitoring using image capturing and analysis system there will be presence of delay in case if there are more number of individuals in a particular area. But one of the main objective functions in the proposed method is to minimize the amount of lag capturing function in the entire system. This lag function is calculated for both captured and non-captured images during time domain representations with a period of shift. Generally the shifting values are considered in order to reduce the dead rate of components in the connected transmission arrangement. For the above mentioned minimization problem only moving individuals are considered in order to avoid unwanted depictions for entire time period. Further the lag functions must be present in lower values which is less than 30 s duration for images that needs to be captured. If the lag period turns above 30 s then images can only be represented in complex transformation matrix and this is processed for separating normal images from non-captured models. Figure 7 denotes the lag time simulation results for moving individuals.

From Fig. 7 it is much clear that lag time which is represented in seconds is much lesser for proposed method as compared to existing method. For real time experimentation total number of moving individuals is represented as 232, 287, 312, 354 and 432 respectively. The above mentioned poignant variations are mostly monitored for changes in time periods between 60 to 360 s. During such movements the lag periods in capturing image segments is equal to 25 and 27 for two distinct moving individuals whereas for remaining three individuals it is represented as 29 s which lies within the defined limit. But in case of existing method no constant lag time is found as more number of images is not captured within the stipulated time period. This can be substantiated for three movement individuals with lag factor of 47, 58, 63 s and after this values only the lag factor decreases to some extent but it crosses the limit that is provided in the reference function as 66 and 68 s respectively. This proves that with minimized lag functions the proposed system can function effectively even at moving intervals.

### Scenario 5

**Figure 8:**
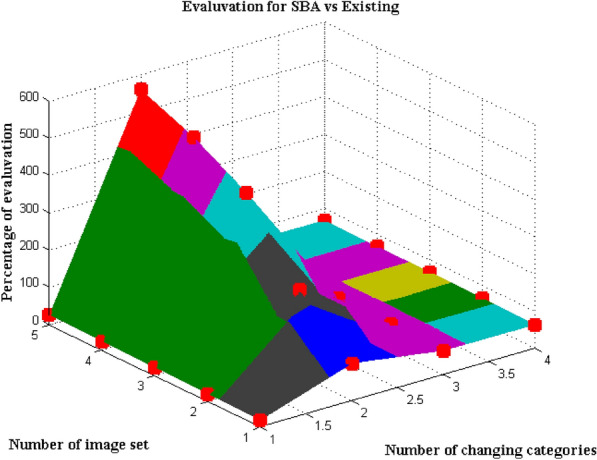
Proportion of estimation with image set.

Image quality factor is one of the important metrics that is used in crowd management system as high image quality provides a clear overview of management procedures to be followed in the mentioned process. As a result the performance analysis of SBA using image quality factor is analyzed in this scenario with changing category depictions and final count is provided by difference in values between reference and current values in data set. This can be represented as a maximization function with separation between image set and changing categories in system model. In a normal process changing categories represents low, medium and high end images that are captured in the multitude and if it is much lower then only minimized values will be provided. Hence to avoid low image capturing system Eq. (8) is formulated and the entire count is provided in the output by continuous loop formation. Figure 8 denotes the simulation results for performance analysis using SBA with total count values.

From Fig. 8 it is clear that number of changing categories is much lesser and it is varied only in the count of 2 or 3 such as 18, 20, 22, 24 and 26 respectively. For each changing categories number of image set is provided as 100, 230, 420, 500 and 560 which denotes that high quality images are provided. For these quality images the percentage of evaluation must be higher and if it above 87 percentage then best performance evaluation is assured for algorithmic cases. The above mentioned performance is achieved in SBA for all image set and changing factors. This can be tested with one small changing factor which is equal to 26 and high quality image that is captured in this case is 560 where during this period the performance evaluation of SBA is equal to 97 percentage. Whereas with same conditions existing method provides performance valuation of 68 percentage which is much lesser than usual performance condition.

## Conclusions

The new path that is used for representing IoT models can be implemented to solve various issues related to many applications. Therefore, a new design model for crowd management is proposed in the article using IoT system integration procedures. Also the projected system aims to solve all complex time domain representations with the presence of transfer function matrix. In addition the major impact that differentiates the proposed model with existing ones is that an analytical model is not provided with derivative functions. Hence the designed system provides a better insight about several viewpoints with optimization algorithm that provides better accuracy. Further one type of neural network algorithm with switching cases is added for changing the individuals from one location to other where three different layers will be used with counting measures. It is always necessary to provide a stable dynamic model in all the places where more number of groups is formed in order to find maximum replication cases using image processing technique. Thus in projected crowd management system a square frame structure is chosen for capturing all images and it is further characterized using green and red color indications. The abovementioned color representation will provide clear information about all the people in the public thus making proper computation using knowledge based representations. After assimilating the proposed model with SBA simulation results are taken for verification and accuracy detection and the same is compared with existing models. From the comparison results it is proved that proposed method which is measured in terms of flow rate, dynamic variations and lag time provides precise outcomes which are more than 83% of existing case studies. In future the crowd management procedure must be processed using robotic technology using artificial intelligence technique.

## Data Availability

The datasets used and/or analyzed during the current study available from the corresponding author on reasonable request.
